# Upper-Limb Movement Quality before and after Surgery in Women with Breast Cancer: An Exploratory Study

**DOI:** 10.3390/s24113472

**Published:** 2024-05-28

**Authors:** Jill Emmerzaal, Nieke Vets, Nele Devoogdt, Ann Smeets, An De Groef, Liesbet De Baets

**Affiliations:** 1 Department of Rehabilitation Sciences, KU Leuven, 3000 Leuven, Belgium; jill.emmerzaal@mcgill.ca (J.E.);; 2 CarEdOn Research Group, 3000 Leuven, Belgium; 3 Department of Vascular Surgery and Department of Physical Medicine and Rehabilitation, Centre for Lymphoedema, University Hospitals Leuven, 3000 Leuven, Belgium; 4 Department of Oncology, KU Leuven, 3000 Leuven, Belgium; 5 Department of Surgical Oncology, University Hospitals Leuven, 3000 Leuven, Belgium; 6 MOVANT Research Group, Department of Rehabilitation Sciences, University of Antwerp, 2000 Antwerp, Belgium; 7 Pain in Motion International Research Group, 1090 Brussels, Belgium; 8 Pain in Motion (PAIN) Research Group, Department of Physiotherapy, Human Physiology and Anatomy, Faculty of Physical Education and Physiotherapy, Vrije Universiteit Brussel, 1090 Brussels, Belgium; 9 Chronic Pain Rehabilitation, Department of Physical Medicine and Physiotherapy, University Hospital Brussels, 1090 Brussels, Belgium

**Keywords:** breast cancer, upper-limb function, pain, movement quality, accelerometry

## Abstract

(1) Background: This study aimed to describe upper-limb (UL) movement quality parameters in women after breast cancer surgery and to explore their clinical relevance in relation to post-surgical pain and disability. (2) Methods: UL movement quality was assessed in 30 women before and 3 weeks after surgery for breast cancer. Via accelerometer data captured from a sensor located at the distal end of the forearm on the operated side, various movement quality parameters (local dynamic stability, movement predictability, movement smoothness, movement symmetry, and movement variability) were investigated while women performed a cyclic, weighted reaching task. At both test moments, the Quick Disabilities of the Arm, Shoulder, and Hand (Quick DASH) questionnaire was filled out to assess UL disability and pain severity. (3) Results: No significant differences in movement quality parameters were found between the pre-surgical and post-surgical time points. No significant correlations between post-operative UL disability or pain severity and movement quality were found. (4) Conclusions: From this study sample, no apparent clinically relevant movement quality parameters could be derived for a cyclic, weighted reaching task. This suggests that the search for an easy-to-use, quantitative analysis tool for UL qualitative functioning to be used in research and clinical practice should continue.

## 1. Introduction

Breast cancer is the most common cancer among women [1]. Although survival rates are high, many women experience persistent physical problems following treatment [2,3,4]. These involve upper-limb (UL) problems (e.g., movement alterations; strength deficits; and altered sensations and pain in the arm, hand, and shoulder), all negatively affecting UL functionality [4]. UL dysfunctions are common and considered normal in the short term after treatment while tissues are healing. However, persistent UL dysfunctions are reported in a subgroup of breast cancer survivors in the long term, even when acute side effects related to tissue healing should have been resolved [4]. These persistent, unhelpful UL dysfunctions clearly interfere with women’s ability to perform tasks of daily living and decrease their quality of life [2,4,5,6].

With the long-term aim to improve UL recovery trajectories in women following breast cancer treatment, the first step is to enable a quantitative assessment of UL functioning during the different recovery stages [7,8]. Such a quantitative assessment can include both high-dimensional data (such as human kinematic or kinetic movement data) and low-dimensional data (such as patient-reported outcomes measures (PROMs)) [9]. Human movement analysis allows for the quantification of altered movement patterns and can be used to evaluate musculoskeletal conditions and function. Altered movement patterns in breast cancer patients reported up to now include specific changes in scapulothoracic kinematics, spatiotemporal movement characteristics, and scapular muscle activation patterns [2]. However, a pitfall of these techniques is that they often require a laboratory setting, limiting their accessibility. PROMs, on the other hand, are easy to use and accessible and can be easily implemented in clinical practice, but they can be subject to ceiling effects and may be affected by reporting bias [9,10]. Although both types of analysis seem complementary to understanding UL function, the use of movement analyses in the breast cancer population is less established compared to PROMs [2].

To develop a quantitative UL functioning analysis for clinical screening and research, there is a need for low-cost tools. Wearable inertial sensors might offer potential as a solution, as they can calculate features beyond established kinematic parameters derived from traditional movement analysis [9,11]. Moreover, a single accelerometer on the lower arm may provide insights into the quality of movement of the complete UL, which is, to the best of our knowledge, currently missing in research on shoulder pathologies [11].

Such dedicated accelerometer-driven measures have been used previously to assess movement quality (in terms of regularity, predictability, smoothness, and stability) [12] and have been described in a range of clinical populations during a habitual task such as running or gait analysis [13,14,15,16,17,18,19,20,21,22,23,24]. Within the field of UL function, these parameters are most often used in individuals with neurological disorders, for example, to analyze the spontaneous arm movement of premature infants with traumatic brain injury [25] or to assess movement smoothness in people post stroke [26,27].

Given all this, the aim of this study is to identify clinically relevant movement quality parameters derived from acceleration signals of one wrist-worn sensor during a reaching task in women who have undergone breast cancer surgery. This exploratory study will compare upper-limb movement quality before and after surgery, as well as the relationship between post-surgical movement quality and self-reported UL disability and pain severity. The results of this study may inform the selection of parameters that are relevant to quantitatively describing UL function following breast cancer surgery as assessed by an easy-to-use accelerometry tool.

## 2. Materials and Methods

### 2.1. Study Participants

This study was part of the UPLIFT-BC project, which is a prospective study investigating the prognostic factors for persistent UL dysfunction in breast cancer survivors. Ethical approval was obtained from the Ethical Committee of the University Hospital Leuven (S66248). For this study, the first 30 participants of the larger project who met the eligibility criteria for this exploratory study were included. Inclusion criteria were (1) women scheduled to undergo unilateral breast-conserving surgery or mastectomy combined with a sentinel lymph-node biopsy or axillary lymph-node dissection for primary breast cancer, (2) a preoperative baseline and one post-op follow-up measurement, and (3) no baseline self-reported disability (i.e., Quick DASH score < 15) [28]. Recruitment and screening of eligibility for all participants were carried out by N.V. All participants provided written informed consent before the start of the study.

### 2.2. Data Acquisition

**Figure 1:**
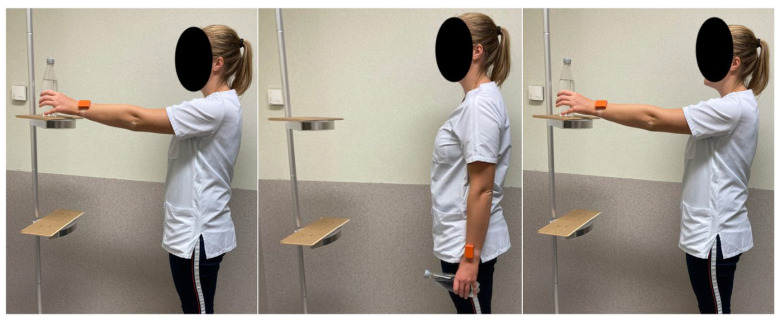
Movement of the object from shoulder level to the side of the body and back to shoulder level. Women were instructed to perform the movement at their preferred speed and in their habitual way. Accelerometer data derived from the sensor located at the distal end of the forearm on the operated side served as input for defining movement quality parameters.

The assessment involved an instrumented cyclic, weighted reaching task that consisted of participants repeatedly moving a 0.5 kg weight from the side of the body to a plate positioned at 90° humerothoracic elevation and back 14 times at a consistent pace while continuing to hold the weight (Figure 1).

It was a distinct choice to use this repeated weighted elevation task to 90° humerothoracic elevation. This movement is needed in many tasks of daily living and can be performed by the different subgroups within the population of breast cancer survivors (with (out) pain, with fear, lymphedema, etc.) in the short term after surgery, as well as in long-term follow-up. Nevertheless, this task also requires neuromuscular coordination and movement at the level of the glenohumeral and scapulothoracic joints [29]. Women were instructed to perform the movement as they would normally (non-constrained movement).

Raw 3-dimensional (3D) accelerometer, 3D gyroscope, and magnetometer data were collected at a rate of 60 Hz using one inertial measurement unit (MVN BIOMECH Awinda, Xsens Technologies, Enschede, The Netherlands) [30]. Data were transmitted to the computer using the Xsens real-time streaming protocol [30].

In line with the recommendations of Xsens, the IMUs were first placed on the upper body of the participants (head, bilaterally at the scapulae, upper arms, lower arms, and hands). Then, the calibration procedure defined by Xsens was performed, which requires all sensors of the upper body to be placed. The calibration procedure consisted of the following steps: standing still in the neutral position for a few seconds, then shortly walking around, subsequently returning to the initial place, and standing still in the neutral position until the calibration procedure was finished. The neutral position is described as the head positioned straight ahead, shoulder girdle relaxed, hands on the lateral thighs with the thumbs pointing forward, and feet hip-width apart with the toes pointing to the front.

For this study’s purpose, only the accelerometer and gyroscope data of the sensor located at the distal end of the forearm on the operated side were considered. The sensor was attached using double-sided tape. A Velcro strap was also placed around the wrists to secure the sensors and minimize excess movement artefacts.

The two assessments were conducted at the Department of Physical Medicine and Rehabilitation of the University Hospitals Leuven at baseline, i.e., 1 week before surgery, and at follow-up, i.e., 3 weeks after surgery. At both assessment moments, participants also filled out the Quick Disabilities of the Arm, Shoulder, and Hand (Quick DASH) questionnaire, which measures self-reported UL disability on a scale of 0 to 100. To score pain severity in the shoulder, arm, or hand region, we used item 9 of the Quick DASH, which scores pain severity on a scale of 1 (no pain severity) to 5 (extreme pain severity). The Quick DASH has adequate measurement properties in women with breast cancer and in breast cancer survivors [31,32].

### 2.3. Data Analysis

We processed and analyzed all data using custom MATLAB scripts (MATLAB 2022b, The Math Works, Inc., Natick, MA, USA). Upon request, more details on the data-analysis method than described below can be provided by the corresponding author.

We filtered the angular velocity signals using a 4th-order forward–backward Butterworth low-pass filter with a cut-off frequency of 2 Hz to identify non-moving data points before and after each repetition. This allowed us to pinpoint the indices of the start and end of the repetitions while retaining only movement data. These indices were then used to segment the raw, unfiltered acceleration signals, which were concatenated back into a single trial without any unintended non-moving periods. Given that 14 repetitions were performed, roughly 1400 data points were available for analyses [33,34].

We derived the following movement quality parameters from the trimmed and concatenated Euclidian norm (2-norm) of the raw acceleration signals (i.e., Pythagorean Theorem over acceleration in x, y, and z directions since no axis is expected to stay stable during movement execution): (1) local dynamic stability, (2) movement predictability, (3) movement smoothness, (4) movement symmetry, and (5) movement variability. The Euclidian norm is the Pythagorean Theorem applied to the time series, over which we calculated the parameters. A detailed description of these parameters is given below.

(1) Local dynamic stability

To quantify local dynamic stability, we utilized the maximum Lyapunov exponent as a measure of a system’s sensitivity to initial conditions and ability to cope with small perturbations [35]. In this study, the system under examination was the movement of the UL. The Lyapunov exponent was calculated by analyzing the rate of increase in the distance between two nearby points in the system over time. A rapid increase in this distance indicates instability in the system [36]. To determine the dominant frequency of the signal, we applied power spectral analysis and used this information to set the range for the calculation of the Lyapunov exponent. We then employed MATLAB’s built-in functions to reconstruct the number of state spaces and time lag that accurately captured the signal and calculated the Lyapunov exponent using Rosenstein’s method and the predetermined parameters [35]. This individualized approach ensured that the time range, number of state spaces, and time lag were specifically tailored for each participant.

(2) Movement predictability

To quantify movement predictability, we employed sample entropy, a mathematical algorithm that assesses the similarity of patterns within a data set [33]. Specifically, we utilized the non-linear mathematical algorithm described by Richman and Moorman (2000) [37]. Sample entropy examines the similarity of patterns within a data set and uses this information to determine the predictability of the data [12]. A data set with high sample entropy indicates a higher level of irregularity or unpredictability, while a data set with low sample entropy indicates low levels of irregularity or predictability. As input parameters for the calculation of sample entropy, we used a sample length of 14 repetitions, a series length (m) of 2 data points, and a tolerance window (r) equal to 0.2 times the standard deviation of the signal at baseline [33]. This person-specific tolerance window was then applied at the follow-up time point.

(3) Movement smoothness

To evaluate movement smoothness, we used the log dimensionless jerk, as proposed by Melendez-Calderon et al. (2021) [27]. The log dimensionless jerk is a measure of the rate at which an object’s acceleration changes over time, calculated as the logarithm of the ratio of the change in acceleration to the change in time. For instance, if an object is accelerating at a constant rate, the log dimensionless jerk would be zero. In contrast, if the object’s acceleration is increasing or decreasing rapidly, the log dimensionless jerk would be higher. The log dimensionless jerk is used as a measure of movement smoothness, and an unsmooth movement pattern is characterized by a high value of log dimensionless jerk.

(4) Movement symmetry

Movement symmetry was defined using the autocorrelation of the signal [15]. As this is a pseudo-cyclic movement, the acceleration signal should repeat itself with a certain time delay. Therefore, the autocorrelation will yield peak values at a time lag equivalent to the period of the signal. The height of the first dominant peak indicates the autocorrelation coefficient of consecutive repetitions with perfect symmetry being equal to one.

(5) Movement variability

Movement variability was quantified by the root mean square (RMS) [38]. RMS is a statistical measure calculated by taking the square root of the mean of the squares of the individual data points within the signal. A high RMS value indicates a substantial amount of movement variability, whilst a low RMS value indicates minimal movement variability. The RMS value of the acceleration signal was calculated for each trial with a sliding window of 10 samples, providing an overall measure of movement variability during the task.

The specific functions applied for each utilized measure per movement quality parameter can be found at https://github.com/movementquality (accessed on 1 March 2024).

### 2.4. Statistical Analysis

Visual inspection of the histogram and Q-Q plots of the data showed a non-normal distribution for most of our parameters. Therefore, we used non-parametric variants of the applied statistical tests. To compare baseline values with follow-up values, we used the Wilcoxon signed rank test as a repeated measures test. To relate self-reported UL disability and UL pain severity to movement quality at the post-surgical time point, we used Spearman’s Rho rank correlation coefficients. The strength of the correlation was defined as follows: 0.00 to 0.25 as little to no relationship, 0.25 to 0.5 as a fair relationship, 0.5 to 0.75 as moderate to good, and 0.75 to 1 as a good-to-excellent relationship [39]. Alpha was set at 0.05 for both analyses. All statistical analyses were performed in Python Notebook SciPy’s stats libraries (version 1.10.0).

## 3. Results

The baseline characteristics of the women (*n* = 30) included in this study can be found in Table 1.

### 3.1. Comparison of Movement Quality Parameters between Pre- and Post-Surgery

**Figure 2:**
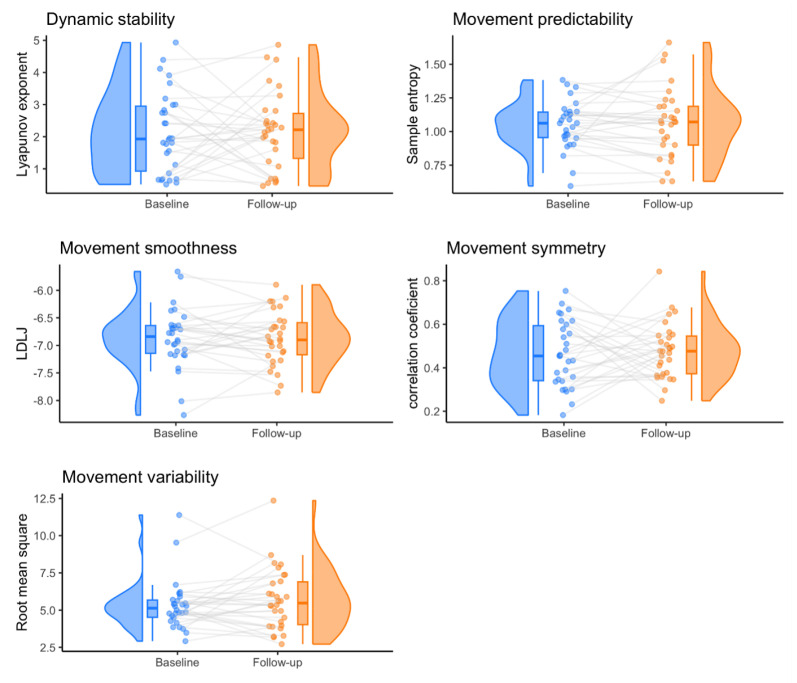
Comparison of movement quality before (baseline) and after (follow-up) surgery represented in half violin plots integrated with box plots and strip plots, together forming raincloud plots. For transparency in data visualization, every dot is an individual person.

Figure 2 shows the pre-surgery and 3-week post-surgery results for the various movement quality parameters. At both time points and for all parameters, raincloud plots that show the distribution of the parameters with a box plot are seen [40,41]. No significant differences between the pre-and post-surgical assessments were observed for any of the movement quality parameters. Figure 2 shows half violin plots with integrated box plots and strip plots, together forming a raincloud plot [40,41]. Here, a highly diverse individual pattern in movement quality parameters from pre- to post-surgery is observed.

### 3.2. Relationship between Self-Reported UL Disability and Movement Quality

**Figure 3:**
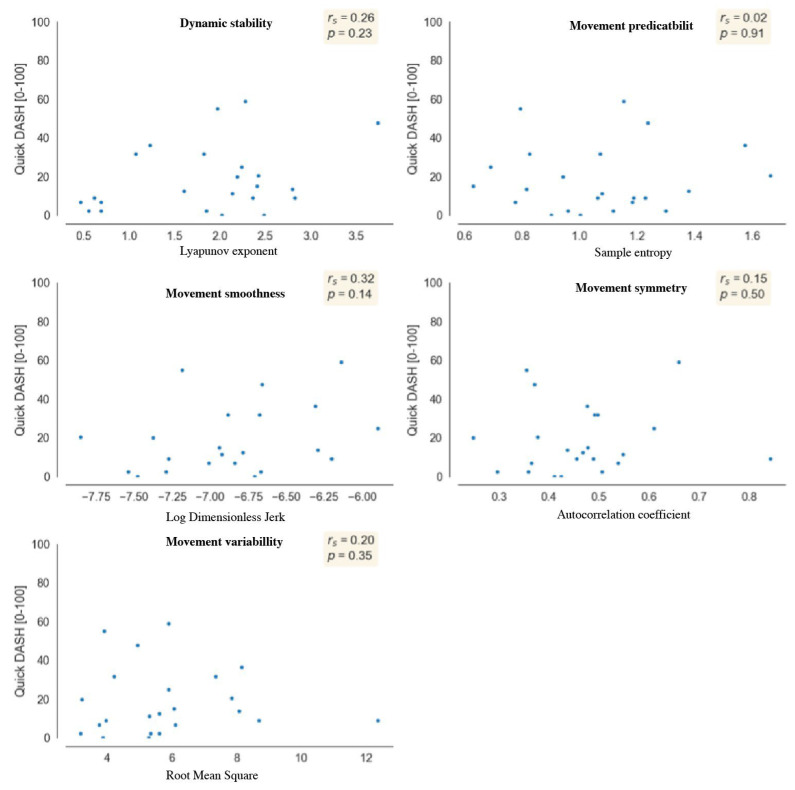
Correlation between self-reported disability and movement quality, where rs = Spearman’s correlation coefficient, and *p* = significant value.

We found positive fair correlations between disability and dynamic stability (rs = 0.26) and between disability and smoothness (rs = 0.32) (Figure 3), indicating that higher levels of self-reported disability are associated with a movement pattern that is less dynamically stable with fewer fluctuations in the acceleration signal (i.e., smoother). However, these fair relationships were not significant (*p* > 0.05). Furthermore, we found few to no relationships between disability and predictability (rs = 0.02), disability and symmetry (rs = 0.15), and disability and variability (rs = 0.2) (Figure 3).

### 3.3. Relationship between Self-Reported UL Pain Severity and Movement Quality

**Figure 4:**
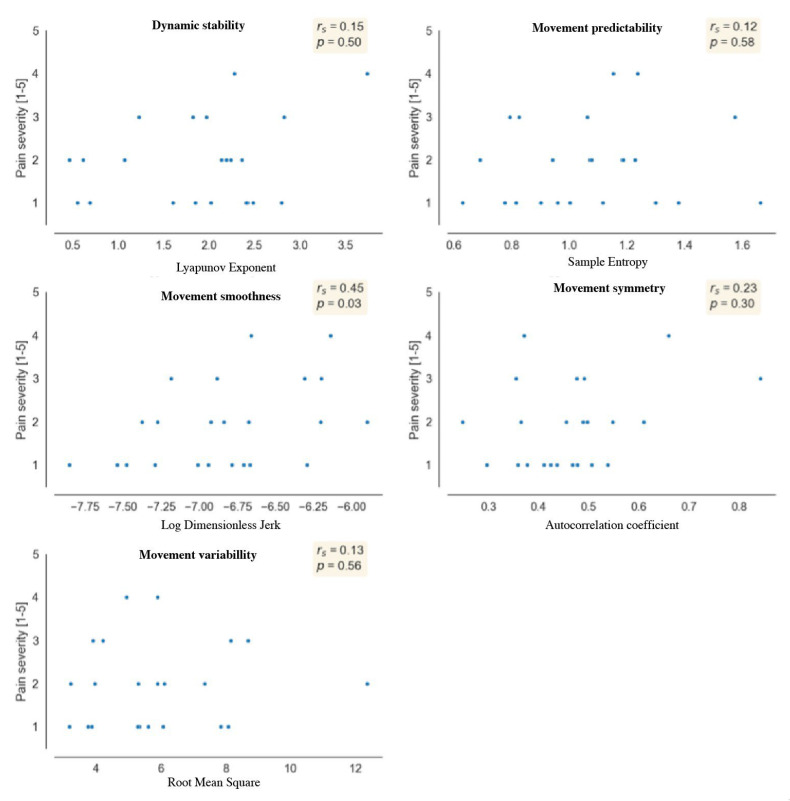
Correlation between self-reported pain severity and movement quality, where rs = spearman’s correlation coefficient, and *p* = significant value.

We did find a significant fair positive relationship between pain severity and movement smoothness (rs = 0.45, *p* = 0.03), indicating that more pain is associated with a smoother movement pattern. Few to no relationships were found between pain intensity and all other parameters (Figure 4).

## 4. Discussion

To the best of our knowledge, this is the first study investigating UL movement quality in women treated for breast cancer via an easy-to-use, low-cost, and accessible measurement set-up, namely a wrist-worn accelerometer, during a reaching task. We were unable to find any significant differences in movement quality pre- versus early post-breast cancer surgery within our study sample, nor in the relation between movement quality and self-reported perceived disability. Except for a single significant relationship between movement smoothness and self-reported UL pain severity, no further relationships were found.

This lack of results is surprising, since the assessed movement quality parameters were found to be sensitive to minute changes in movement patterns in various populations [12]. For example, early gait and balance adaptations could be detected in individuals with multiple sclerosis, as well as in pre-manifesting Huntington’s disease [22,42]. This highlights the fact that knowledge from lower-limb movement quality cannot easily be transferred to the upper limbs, given their more complex movement behavior with a higher number of degrees of freedom and more non-cyclic movement.

There are several methodological considerations that might explain why we did not find any significant results. First, we used a cyclic, weighted reaching task that we did not constrain, and our participants were unfamiliar with this movement task. Task familiarity might be an important aspect in studying non-linear dynamics and explain why we found no differences [26]. In previous research, all the parameters were predominantly used during habitual activities (e.g., walking, running, keeping balance, or spontaneous movements). Movement smoothness is considered a measure of well-trained motor behavior [26], and this theory might be extrapolated to the other movement quality parameters. Therefore, a possible explanation may be that the movement task applied in this study is potentially not suitable for these types of analysis. Second, available literature on these movement quality parameters in biomechanics mainly concerns the lower-limb region or the UL region of subjects with neurological disorders, e.g., stroke in premature infants [26]. The spontaneous arm movement of infants with brain injuries was found to be more unstable (larger Lyapunov exponents) and more unpredictable (larger sample entropy values) than that of those without brain injury [26]. This indicates that these parameters can be successfully applied to the upper extremities. However, to the best of our knowledge, no information on movement quality in other shoulder pathologies has been reported in other investigations.

Although only a single significant correlation was found, some trends in the data were noticed. The fair positive correlations of dynamic stability, smoothness, and symmetry with UL pain severity suggest that women who experience more pain have a more unstable and smoother and symmetrical movement pattern. Similarly, for the positive fair correlations between self-reported UL disability and dynamic stability and smoothness, women who report a higher level of UL disability also tend to show a more unstable and smoother movement pattern. The smoother movement pattern that is associated with more disability and more severe pain may be explained by the fact that women without disability and pain are faster in their deceleration and acceleration at the endpoints [26]. Women with more disability might be more cautious, which mimics a smoother movement. However, this should be further investigated. These trends suggest that there may be relationships between movement quality, UL disability, and pain; however, due to the small size of the study sample, these results should be interpreted with caution. Therefore, we believe that a better result might be found with a movement task that people are familiar with or that elicits spontaneous movement rather than making the task more complex, as described previously in the discussion. Further research with larger sample sizes is necessary to provide stronger information on these trends and their clinical relevance.

Using a habitual task might also provide the solution for another methodological consideration with these non-linear analyses. To obtain accurate results from non-linear analysis, you would need a large data set. Our repetition count (i.e., 14 reps or roughly 1400 data points) is based on the papers by Yentes et al. [33] and TenBroek et al. [34]. They showed that for sample entropy and the Lyapunov exponent, a minimum of 200 and 1500 samples are needed, respectively, but preferably more. While the calculation of sample entropy with 200 samples was possible, unpublished data from Yentes’ lab show that the parameter stabilizes after 2000 samples [33]. For our study, the number of data points captured by the IMU was assumed to be a number of samples similar to those reported by Schutte [43] and Emmerzaal [13] in running and gait biomechanics, respectively. In contrast, Yentes et al. [33], who calculated sample entropy based on step length, step width, and step time parameters, interpreted one step length/width/time as a single sample. This means that the number of steps taken in their study was 200, and furthermore, they showed that it stabilizes after 2000 steps. While sample entropy can be calculated based on any time series [37], the interpretability of the results would change. Metrics calculated based on temporal data are more easily interpreted than those calculated based on acceleration data. However, obtaining the quantity of data required to calculate non-linear dynamics based on UL temporal parameters using a weighted cyclic reaching task is impossible. Therefore, using a habitual and spontaneous arm movement (e.g., arm swing during walking) to capture that amount of data might be more realistic and achievable, especially when gait is not impaired.

Based on our results, we recommend that the search for an easy-to-use, quantitative analysis tool for UL functioning to be used in clinical screening and research continue. This is with the aim of better understanding UL problems after breast cancer to optimize function and improve health outcomes for women with persistent UL dysfunction after breast cancer. Based on studies in other populations [22,25,26,42,44] and the results of this exploratory study, it seems relevant to gather these insights on movement quality, but a repeatable, habitual activity should be used.

## 5. Conclusions

No differences in UL movement quality before and after breast cancer surgery were found for a cyclic, weighted reaching task. This might be due to the highly diverse individual pattern observed in movement quality parameters from pre- to post-surgery. We found fair positive correlations of dynamic stability, smoothness, and symmetry with UL pain severity, suggesting that women who experience more pain have a more unstable, smoother, and more symmetrical movement pattern. Similarly, for the positive fair correlations between self-reported UL disability and dynamic stability and smoothness, women who report a higher level of UL disability also tend to show a more unstable and smoother movement pattern. However, most of these correlations were not significant and should, thus, should be interpreted with caution. We recommend continuing the search for an easy-to-use tool to quantitatively describe UL movement quality. In this regard, a habitual movement task may be more suitable, both for task familiarity and to more easily obtain large quantities of data.

## Figures and Tables

**Table 1:** Participants’ baseline characteristics.

Characteristics	*n* (%) or Mean ± std ^1^
Age (years)	54.27 ± 11.43
BMI (kg/m^2^)	24.61 ± 4.27
Type of Surgery	
• Mastectomy	12 (40%)
• Breast-conserving surgery	18 (60%)
• Sentinel lymph-node biopsy	23 (77%)
• Axillary lymph-node dissection/axillary node clearance	6 (23%)
Surgery on dominant side	8 (27%)
Adjuvant chemotherapy	8 (27%)
Pre-surgery Quick DASH ^2^ score	3.35 ± 4.36
Post-surgery Quick DASH ^2^ score	18.59 ± 17.44
Pre-surgery pain severity	1.1 ± 0.3
Post-surgery pain severity	1.9 ± 1.0

^1^ Standard deviation. ^2^ Disability of Arm, Shoulder, and Hand questionnaire.

## Data Availability

The data that support the findings of this study are not openly available due to GDPR guidelines (i.e., human motion data) and are available from the corresponding author upon reasonable request.
